# Individual variability in diving, movement and activity patterns of adult bearded seals in Svalbard, Norway

**DOI:** 10.1038/s41598-018-35306-6

**Published:** 2018-11-19

**Authors:** Charmain D. Hamilton, Kit M. Kovacs, Christian Lydersen

**Affiliations:** Norwegian Polar Institute, Fram Centre, N-9296 Tromsø, Norway

## Abstract

Bearded seals are one of the least studied Arctic marine mammals, despite their circumpolar distribution and importance as a resource to Inuit communities. In this study, adult bearded seals (*Erignathus barbatus*) were equipped with GPS-Argos-CTD-SRDLs in Svalbard, Norway (2011–2012, n = 7) to document their diving, activity and movement patterns in a region where their habitat is changing rapidly. Five seals transmitted for > 8 months, sending 21,738 GPS-positions and 17,866 dives between July and April. The seals spent little time hauled out (≤ 5%). Diving, which occupied 74 ± 3% of their time, was generally shallow (24 ± 7 m, max: 391 m) and of short duration (6.6 ± 1.5 min, max: 24 min) with deeper, longer dives in winter/spring compared to summer. All seals occupied shallow, coastal areas and relatively small 50% home ranges (10–32 km^2^). However, individuals exhibited high degrees of specialization in their habitat use and diving behaviour, differing markedly with respect to proportions of benthic vs pelagic dives (range: 51–95% benthic dives), distance to glacier fronts (range: 3–22 km) and in the time spent at the bottom of dives (range: 43–77%). Having specialized strategies within a generalist population may help bearded seals adapt in a rapidly changing Arctic ecosystem.

## Introduction

The Arctic is being impacted by climate change more rapidly than other areas of the globe. Air temperatures are increasing at a rate three times greater than the global average and Arctic sea-ice extent is declining rapidly^1^. Numerous effects of climate change have been documented in both terrestrial and marine ecosystems^2–5^. Arctic endemic marine mammals will likely be impacted significantly by climate change induced alterations to their habitats because of their generally high trophic level positions, long life-cycles compared to the rapid pace at which change is occurring, limited dispersal options and tight associations with sea ice^3,6^. Impacts will differ among species depending on the nature of their relationships to sea ice, sensitivity to the changes that are taking place and their level of behavioural plasticity^6,7^. The level of naturally occurring variation within traits among individuals will also likely be highly relevant, as high levels of variation may help species adapt faster to changing conditions^3,8,9^.

Bearded seals (*Erignathus barbatus*) are an Arctic endemic species with a patchy, circumpolar distribution^10^. This large true seal generally feeds on benthos (infauna, epifauna and benthically distributed fishes and invertebrates) in relatively shallow, coastal areas^11–14^. They are associated with drifting sea ice throughout the year, using ice (either the land-fast ice edge or drifting sea ice) as a haul-out platform year-round and also as a birthing and nursing platform in the spring^12,13,15–17^. Bearded seals generally use ice in areas that have natural holes and leads for breathing, although they do make and maintain breathing holes in thin ice in some parts of their range^11–13,18^. Bearded seals are important prey for polar bears (*Ursus maritimus*) and are an important subsistence resource for Arctic communities in many parts of their range^10,19^. Despite their broad distribution across the Arctic and their importance for subsistence communities, relatively little is known about this species. Some biotelemetry data from bearded seals in Svalbard, Norway has been published, but it is mainly limited to data from pups (VHF telemetry and time-depth recorder data); information on adults is limited to tracking animals within short seasonal time frames^20–24^. Beyond Svalbard the only published biotelemetry data from bearded seals focusses on habitat selection of young (< 2) bearded seals and methodological development of models to describe animal movements^17,25^.

**Figure 1:**
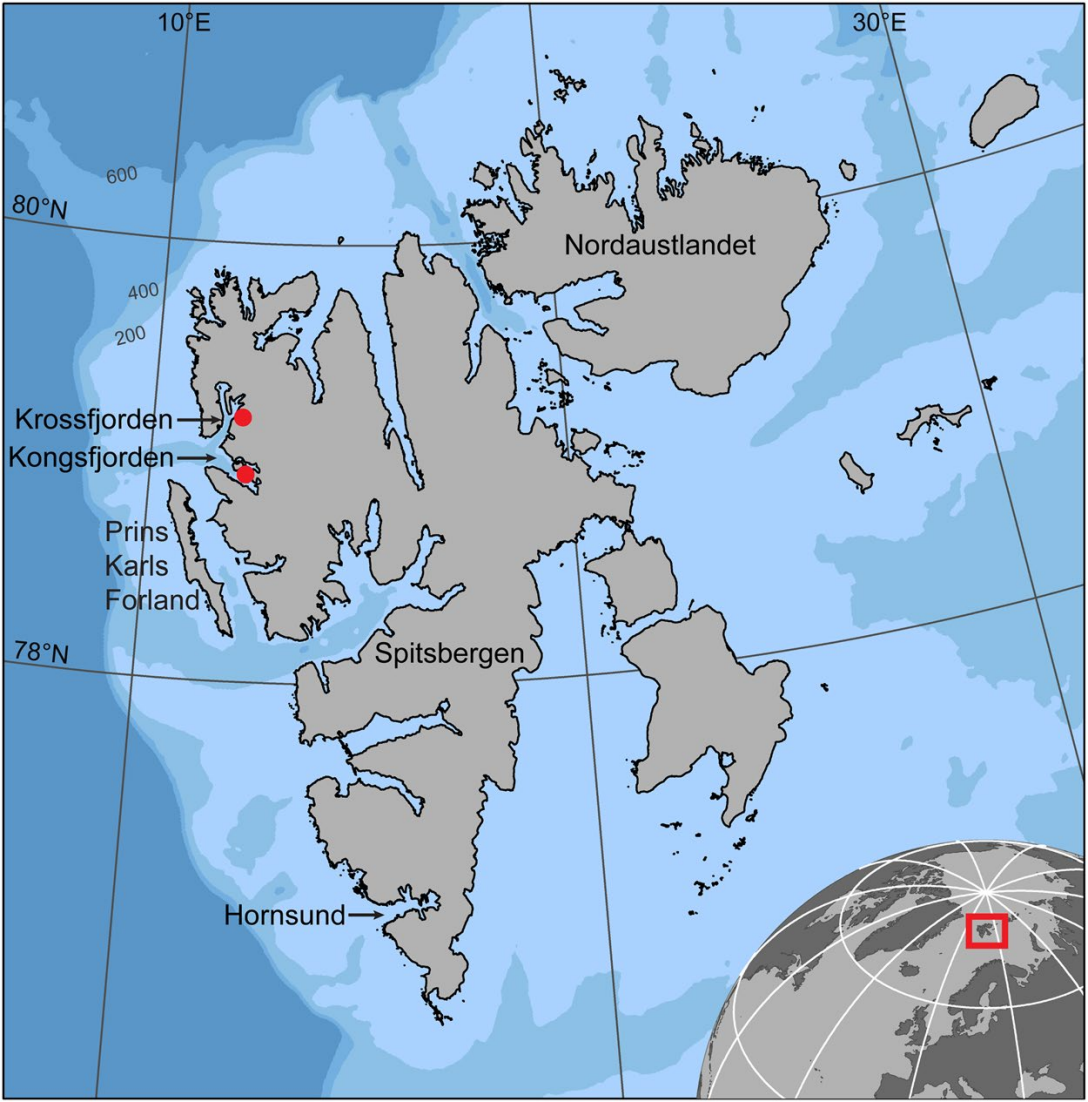
Map of Svalbard. Map of the Svalbard Archipelago showing place names, bathymetry and tagging locations (red circles) for seven adult bearded seals (*Erignathus barbatus*) equipped with GPS Argos CTD Satellite Relay Data Loggers in 2011–2012.

Bearded seals are distributed at low densities throughout fjords and in some coastal shelf areas in the High-Arctic Archipelago of Svalbard (10–35°E, 74–81°N; Fig. 1). Svalbard is an Arctic hot-spot that is warming at a faster rate than other Arctic areas. This region has had the largest decline in the seasonal duration of sea-ice cover and the fastest rates of air temperature increase in the European Arctic^26,27^. The amount of land-fast ice formation in Svalbard fjords has also decreased greatly in recent years^28^. These changes in sea ice are due in part to Atlantic Water intruding more frequently across the polar front that forms between the West Spitsbergen Current (i.e. Atlantic Water) and East Spitsbergen Current (i.e. Arctic Water) on the west coast, resulting in increased amounts of Atlantic Water in Svalbard’s fjords^29^.

The potential impacts on bearded seals of the ongoing environmental changes taking place in the Arctic are difficult to address given how little research effort has been directed to this species. The purpose of the present study was to investigate the space use, diving behaviour and movement and activity patterns of adult bearded seals in the context of a rapidly changing environment in the Svalbard area. This was achieved by investigating: home range size; the habitat the seals used; seasonal and diurnal patterns in activity and diving behaviour; and by exploring the variation in diving behaviour and habitat utilization among individual seals. Seals were considered specialists if their behaviour (i.e. use of habitat, diving) was consistent throughout the tracking period and generalists if behaviours varied over time.

## Results

A total of 21,738 GPS locations (daily mean ± SD per seal: 15 ± 6, daily range: 1–35), 17,866 dives (daily mean ± SD per seal: 18 ± 15, daily range: 1–99) and 2,392 CTD profiles (daily mean ± SD per seal: 2 ± 1, daily range: 1–6) were transmitted by the seven instrumented bearded seals (Table 1), which were all adult animals. Five of the seven animals transmitted data from when they were tagged immediately post-moult (late summer) through until the following spring (241–286 d), while the other two had shorter data records (19 and 82 d respectively; Table 1).

**Table 1:** Tagging information for seven adult bearded seals (*Erignathus barbatus*) equipped with GPS Argos CTD Satellite Relay Data Loggers in 2011–2012 in Svalbard, Norway. ID codes identify sex (M for males, F for females) and body mass of the seals.

ID	Sex	Length (cm)	Girth (cm)	Mass (kg)	Date	Latitude (°N)	Longitude (°E)	Duration (d)
F310	F	198	171	310	2011–07–25	79.19	12.14	241
F336	F	200	178	336	2011–07–21	78.92	12.46	286
F340	F	199	180	340	2011-08-06	78.99	12.40	82
F385	F	214	185	385	2011-07-29	78.91	12.48	272
M250	M	180	160	250	2011-07-19	78.96	12.06	285
M280	M	191	165	280	2012-08-14	78.92	12.45	19
M305	M	195	171	305	2012-08-18	78.94	12.30	243

### Movement and home range

**Figure 2:**
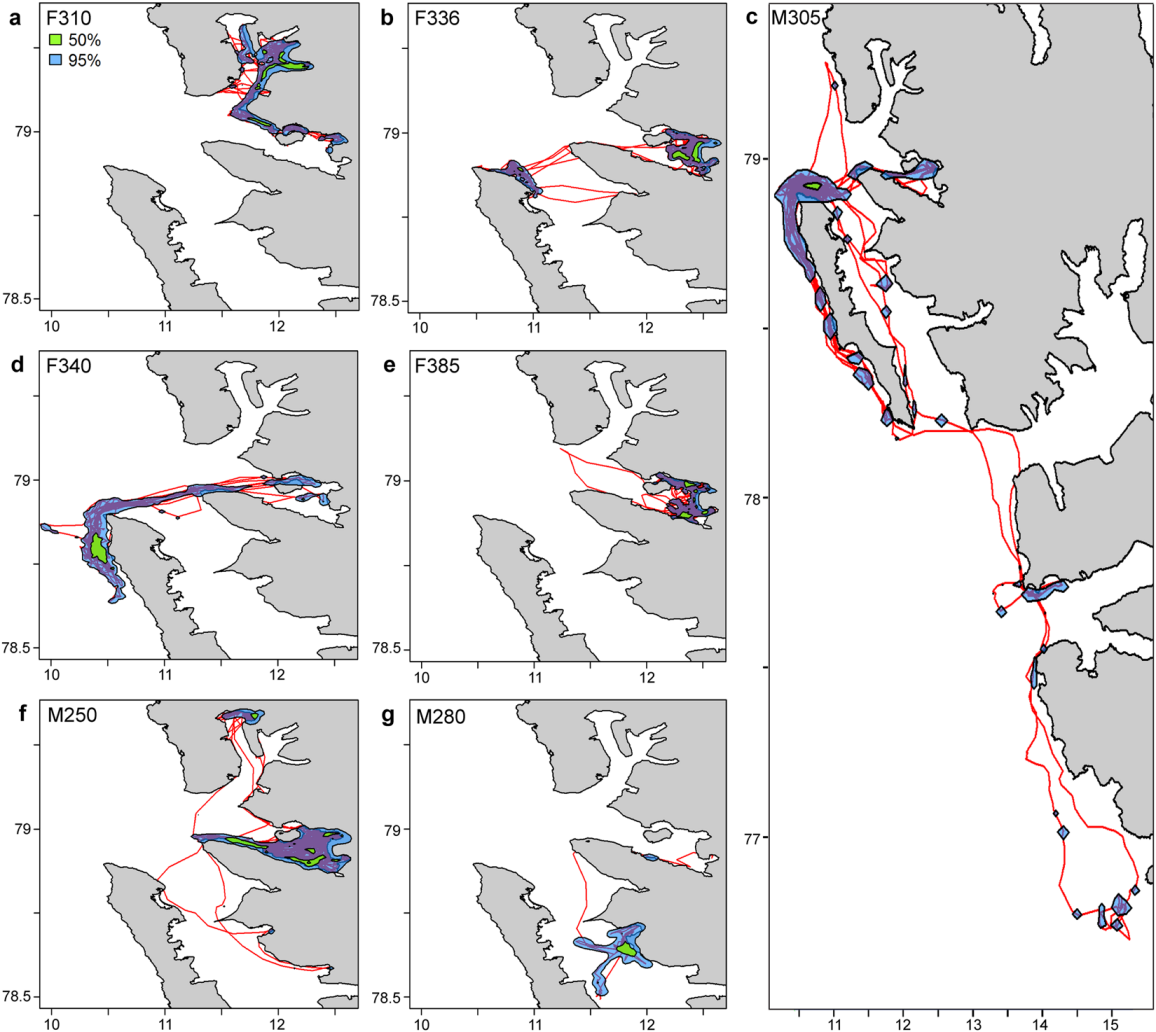
Bearded seal tracks and home ranges. Map of the tracks (red), 50% home range (green) and 95% home range (blue) for seven adult bearded seals (*Erignathus* barbatus) equipped with GPS Argos CTD Satellite Relay Data Loggers in 2011–2012 in Svalbard, Norway.

Four of the seven tagged seals spent the majority of their tracking period within the Kongsfjorden/Krossfjorden complex (Fig. 2). The other three seals focussed their activities around Prins Karls Forland, with two of these seals travelling into Kongsfjorden occasionally to haul out. One seal (M305) travelled much more broadly than the other six. This animal shifted from the area where it was instrumented in Kongsfjorden (79.3°N, 10.9°E) south to the entrance to Hornsund (76.7°N, 15.3°E; a distance of 306 km between its northernmost and southernmost location), stopping en route for days or weeks at a time at a few sites (Fig. 2).

**Figure 3:**
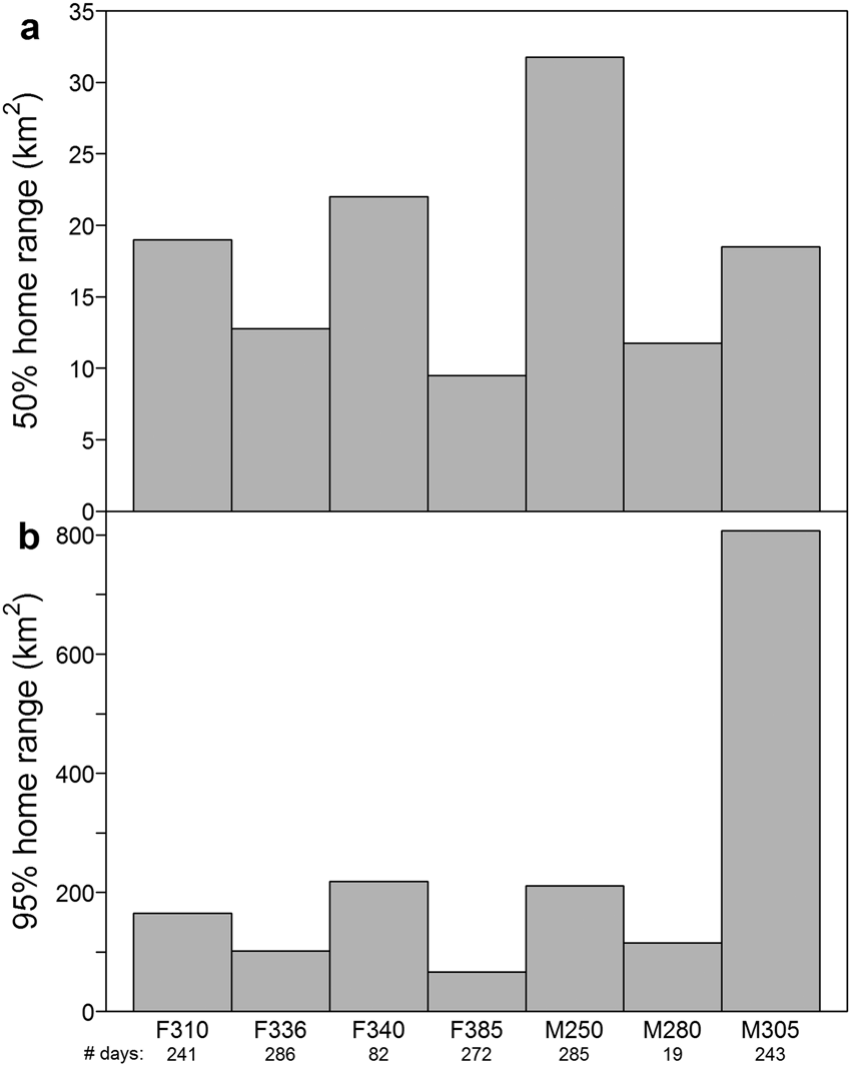
Brownian bridge home range sizes. 50% and 95% brownian bridge home range sizes for seven adult bearded seals (*Erignathus barbatus*) equipped with GPS Argos CTD Satellite Relay Data Loggers in 2011–2012 in Svalbard, Norway. The number of days of data transmission is provided under the seal IDs on the x-axis.

The 95% home ranges for six of the seven seals were between 67 and 218 km^2^, while the 95% home range for M305 was much larger (807 km^2^) (Fig. 3). The 50% home ranges were quite similar among all individuals, varying between 10 and 32 km^2^ (Fig. 3). There were no marked seasonal trends in the size of either the 95% or 50% home ranges (Table 2). In the spring breeding period (April and May), the two male bearded seals had home ranges more than twice the size of the three female bearded seals that were still transmitting data at this time (Table 2).

**Table 2:** Activity and environmental variables (mean ± 95% CI) and 50% and 95% home range size (km^2^) by month for seven adult bearded seals (*Erignathus barbatus*) equipped with GPS Argos CTD Satellite Relay Data Loggers in 2011-2012 in Svalbard, Norway. The p-values (in bold) are the results of linear models testing for a seasonal trend in each variable.

Seal	Month	Time spent diving (%)	Time spent hauling out (%)	Time spent at the surface (%)	Haul-out duration (min)	Interval between haul-out events (h)	Number of haul-out events	Distance to glacier front (km)	Distance to coast (km)	Bathymetric depth (m)	50% home range (km^2^)	95% home range (km^2^)
F310	All	72 (71–74)	6 (5–8)	21 (21–22)	353 (279–426)	84 (53–114)	65	4.7 (4.6–4.8)	0.3 (0.3–0.4)	69 (67–71)	19	165
	Jul	60 (48–72)	23 (9–38)	17 (14–20)	302 (114–492)	5 (0–11)	7	3.5 (2.9–4.0)	0.3 (0.3–0.4)	81 (69–94)	—	—
	Aug	64 (60–69)	16 (10–21)	20 (18–23)	363 (251–474)	30 (16–43)	21	4.1 (3.9–4.3)	0.5 (0.4–0.5)	77 (71–82)	10	85
	Sep	66 (62–70)	6 (2–10)	28 (25–31)	282 (130–433)	78 (52–105)	9	5.8 (5.6–6.0)	0.4 (0.3–0.4)	91 (84–97)	15	117
	Oct	70 (67–74)	6 (2–10)	24 (21–26)	426 (243–607)	109 (0–259)	6	5.2 (5.0–5.4)	0.2 (0.2–0.3)	81 (75–87)	8	65
	Nov	76 (73–80)	4 (1–7)	20 (18–22)	407 (136–678)	132 (87–177)	4	4.7 (4.5–4.8)	0.2 (0.2–0.3)	73 (69–78)	7	48
	Dec	77 (73–80)	5 (2–9)	18 (16–20)	294 (78–508)	106 (3–210)	8	3.6 (3.4–3.8)	0.2 (0.2–0.2)	58 (54–61)	4	33
	Jan	76 (72–80)	5 (2–9)	19 (16–21)	1125 (649–1600)	261 (60–465)	2	4.6 (4.4–4.8)	0.4 (0.3–0.4)	50 (47–54)	8	44
	Feb	77 (74–79)	1 (0–3)	22 (20–24)	203 (0–492)	271 (31–510)	3	4.6 (4.3–4.9)	0.5 (0.5–0.6)	46 (41–50)	14	122
	Mar	78 (76–81)	1 (0–2)	21 (18–24)	257 (139–376)	127 (0–254)	5	5.4 (5.1–5.6)	0.3 (0.3–0.4)	76 (69–82)	5	27
	*p*	**<0.001**	**<0.001**	**0.169**	**0.693**	**<0.001**		**0.771**	**0.001**	**<0.001**	**0.501**	**0.373**
F336	All	72 (71–73)	5 (4–6)	24 (23–24)	421 (321–520)	139 (82–194)	44	5.6 (5.4–5.8)	1.3 (1.2–1.3)	25 (24–26)	13	101
	Jul	56 (47–66)	22 (11–34)	21 (17–25)	366 (133–600)	11 (1–21)	9	4.3 (3.4–5.2)	0.3 (0.2–0.4)	35 (31–39)	—	—
	Aug	74 (70–79)	8 (4–13)	18 (16–19)	686 (282–1098)	134 (0–340)	5	13.0 (12.4–13.7)	1.2 (1.1–1.4)	16 (13–19)	8	184
	Sep	76 (73–79)	4 (1–7)	20 (18–22)	611 (551–670)	246 (0–560)	3	14.8 (14.1–15.5)	2.0 (1.9–2.2)	17 (16–19)	5	45
	Oct	72 (68–75)	5 (2–7)	24 (22–26)	222 (80–365)	72 (27–118)	9	3.2 (3.0–3.4)	1.1 (1.0–1.3)	15 (14–17)	5	30
	Nov	69 (66–72)	5 (1–8)	26 (24–29)	493 (431–554)	168 (0–348)	4	3.4 (3.1–3.6)	1.2 (1.1–1.4)	13 (12–15)	4	27
	Dec	69 (65–72)	2 (0–5)	29 (26–32)	1000 (1000–1000)	528 (528–528)	1	3.8 (3.6–4.0)	1.4 (1.3–1.6)	15 (14–17)	4	29
	Jan	70 (67–73)	4 (0–7)	26 (24–28)	378 (30–724)	195 (41–349)	4	1.8 (1.7–2.0)	0.5 (0.4–0.6)	30 (27–34)	6	37
	Feb	73 (70–76)	3 (0–5)	24 (22–27)	263 (68–456)	126 (10–240)	4	2.6 (2.4–2.8)	1.1 (0.9–1.2)	48 (44–52)	9	48
	Mar	78 (75–80)	3 (0–5)	20 (18–21)	409 (130–693)	332 (33–628)	3	5.1 (4.9–5.2)	2.2 (2.0–2.3)	30 (28–32)	6	29
	Apr	72 (68–75)	3 (0–6)	26 (23–28)	605 (479–731)	166 (95–237)	2	3.5 (3.3–3.6)	1.2 (1.0–1.3)	37 (35–39)	4	23
	*p*	**0.481**	**<0.001**	**<0.001**	**0.919**	**<0.001**		**<0.001**	**0.063**	**0.001**	**0.841**	**0.102**
F340	All	74 (71–77)	5 (3–8)	21 (19–23)	470 (297–640)	139 (0–282)	10	15.9 (15.6–16.3)	2.4 (2.3–2.5)	29 (27–31)	22	219
	Aug	70 (65–75)	7 (2–12)	23 (19–26)	447 (220–672)	38 (0–104)	5	16.6 (15.9–17.3)	2.9 (2.7–3.2)	40 (35–46)	28	192
	Sep	77 (73–81)	2 (0–5)	21 (17–24)	277 (110–443)	167 (0–433)	3	17.0 (16.5–17.5)	2.3 (2.2–2.4)	23 (21–26)	14	105
	Oct	75 (70–80)	6 (1–11)	19 (16–22)	816 (783–850)	299 (0–688)	2	13.5 (13.0–14.0)	2.0 (1.9–2.1)	24 (21–27)	8	137
F385	All	76 (75–77)	4 (3–5)	20 (19–20)	249 (180–317)	101 (62–139)	62	3.0 (2.9–3.0)	0.7 (0.7–0.7)	32 (31–33)	10	67
	Jul	74 (68–81)	1 (0–3)	25 (19–31)	—	—	—	1.4 (1.1–1.7)	0.3 (0.1–0.4)	21 (14–28)	—	—
	Aug	74 (70–78)	9 (4–13)	17 (16–18)	361 (190–533)	61 (22–100)	11	2.0 (1.9–2.1)	0.5 (0.4–0.5)	26 (23–28)	3	19
	Sep	72 (69–76)	7 (3–10)	21 (19–23)	204 (71–338)	51 (28–74)	14	2.0 (1.9–2.1)	0.5 (0.4–0.5)	23 (21–25)	5	23
	Oct	72 (67–76)	7 (3–11)	22 (19–24)	172 (54–291)	37 (16–58)	18	2.1 (2.0–2.2)	0.4 (0.4–0.5)	21 (19–23)	3	18
	Nov	81 (78–83)	1 (0–3)	18 (17–20)	209 (21–391)	112 (69–154)	4	2.6 (2.4–2.7)	0.6 (0.5–0.6)	26 (24–28)	5	33
	Dec	80 (78–83)	3 (0–5)	17 (16–18)	543 (170–917)	361 (48–673)	2	3.3 (3.2–3.5)	0.7 (0.6–0.8)	32 (30–34)	3	15
	Jan	81 (79–83)	2 (0–5)	17 (16–17)	351 (77–623)	240 (0–587)	3	3.8 (3.7–3.9)	0.9 (0.8–1.0)	35 (34–37)	1	17
	Feb	72 (68–76)	2 (0–5)	26 (23–30)	405 (0–938)	292 (56–527)	2	3.1 (2.9–3.3)	0.8 (0.7–0.8)	47 (43–51)	10	55
	Mar	79 (78–81)	1 (0–3)	19 (18–20)	274 (0–622)	465 (167–771)	2	4.4 (4.2–4.6)	1.0 (0.9–1.1)	41 (38–44)	4	52
	Apr	76 (73–78)	2 (0–5)	22 (20–24)	192 (47–335)	127 (0–257)	6	3.6 (3.5–3.8)	1.1 (0.9–1.2)	40 (38–42)	5	22
	*p*	**0.009**	**<0.001**	**0.002**	**0.936**	**0.032**		**<0.001**	**<0.001**	**<0.001**	**0.493**	**0.201**
M250	All	73 (72–74)	6 (5–7)	21 (21–22)	328 (267–390)	94 (65–123)	69	6.8 (6.6–6.9)	0.7 (0.7–0.8)	51 (49–52)	32	211
	Jul	69 (64–74)	5 (1–9)	26 (23–28)	102 (19–185)	5 (2–7)	8	4.1 (3.3–5.0)	0.5 (0.4–0.6)	83 (76–90)	—	—
	Aug	67 (63–70)	8 (4–11)	26 (24–27)	207 (129–286)	56 (8–103)	16	4.5 (4.3–4.8)	0.4 (0.3–0.5)	88 (83–92)	3	320
	Sep	71 (67–75)	9 (5–14)	20 (18–22)	557 (330–783)	87 (15–160)	7	9.4 (9.0–9.8)	0.5 (0.4–0.6)	40 (36–45)	5	59
	Oct	73 (69–76)	3 (0–6)	24 (22–27)	348 (211–484)	156 (122–190)	4	9.8 (9.4–10.2)	0.5 (0.5–0.6)	38 (33–43)	9	89
	Nov	70 (65–74)	11 (6–15)	20 (18–21)	489 (353–626)	82 (31–133)	9	7.6 (7.1–8.1)	1.0 (0.9–1.1)	35 (30–39)	14	108
	Dec	79 (76–82)	2 (0–4)	19 (17–21)	448 (393–504)	233 (230–235)	2	7.3 (6.9–7.8)	0.8 (0.7–0.9)	30 (26–35)	11	97
	Jan	76 (73–79)	3 (1–6)	21 (18–23)	311 (37–588)	174 (27–321)	5	7.5 (7.0–8.0)	0.9 (0.8–1.0)	34 (30–38)	19	109
	Feb	76 (72–80)	2 (0–5)	22 (18–25)	233 (43–423)	140 (0–348)	3	3.8 (3.4–4.2)	0.7 (0.6–0.8)	39 (35–43)	6	56
	Mar	76 (73–80)	5 (2–8)	19 (17–21)	440 (209–669)	183 (21–346)	5	5.9 (5.5–6.2)	0.9 (0.9–1.0)	64 (60–69)	14	78
	Apr	70 (66–75)	9 (4–14)	21 (18–24)	344 (166–521)	82 (23–142)	10	5.2 (4.8–5.6)	1.0 (0.9–1.1)	72 (66–78)	16	94
	*p*	**0.002**	**0.372**	**<0.001**	**0.066**	**0.009**		**<0.001**	**<0.001**	**0.139**	**0.061**	**0.153**
M280	Aug	71 (66–76)	8 (3–14)	21 (18–23)	205 (77–334)	36 (0–79)	9	6.2 (5.7–6.8)	1.6 (1.4–1.8)	45 (41–49)	-	-
M305	All	79 (77–80)	4 (2–5)	18 (17–18)	516 (358–675)	253 (0–578)	22	21.7 (21.5–21.9)	3.6 (3.5–3.7)	27 (25–28)	19	807
	Aug	69 (63–76)	9 (3–15)	21 (18–25)	308 (219–397)	36 (0–94)	5	15.7 (14.6–16.9)	2.4 (2.2–2.7)	29 (24–34)	-	-
	Sep	68 (63–73)	10 (5–16)	21 (19–24)	633 (262–1006)	41 (0–110)	6	20.2 (19.6–20.9)	3.6 (3.4–3.7)	25 (22–27)	7	62
	Oct	79 (78–81)	0 (0–0)	21 (19–22)	—	—	—	21.6 (21.4–21.7)	3.0 (2.9–3.1)	13 (13–14)	7	31
	Nov	81 (79–83)	0 (0–0)	19 (17–21)	—	—	—	23.1 (22.9–23.3)	3.4 (3.2–3.5)	19 (18–20)	8	44
	Dec	82 (80–84)	0 (0–0)	18 (16–20)	—	—	—	23.5 (23.1–23.9)	3.2 (3.1–3.4)	22 (21–23)	11	52
	Jan	83 (81–86)	1 (0–2)	16 (14–18)	310 (310–310)	3474 (3474–3474)	1	21.2 (20.6–21.8)	3.2 (3.0–3.4)	32 (28–36)	36	586
	Feb	74 (69–79)	11 (6–17)	15 (13–16)	629 (327–926)	22 (4–41)	7	24.0 (23.4–24.6)	7.0 (6.3–7.7)	51 (45–58)	22	446
	Mar	84 (83–86)	1 (0–2)	15 (14–16)	290 (290–290)	944 (944–944)	1	22.9 (22.2–23.5)	2.9 (2.8–3.1)	25 (21–28)	11	235
	Apr	80 (74–86)	6 (0–13)	14 (13–16)	502 (0–1004)	180 (0–417)	2	18.3 (17.3–19.2)	3.1 (2.9–3.3)	29 (25–33)	—	—
	*p*	**<0.001**	**0.085**	**<0.001**	**0.732**	**0.069**		**<0.001**	**<0.001**	**<0.001**	**0.251**	**0.115**
Mean	All	74 (72–76)	5 (4–6)	21 (20–22)	363 (282–442)	121 (74–167)	40 (21–58)	9.1 (4.4–14.0)	1.5 (0.7–2.3)	40 (29–50)	18 (13–23)	241 (61–414)

### Habitat use

**Figure 4:**
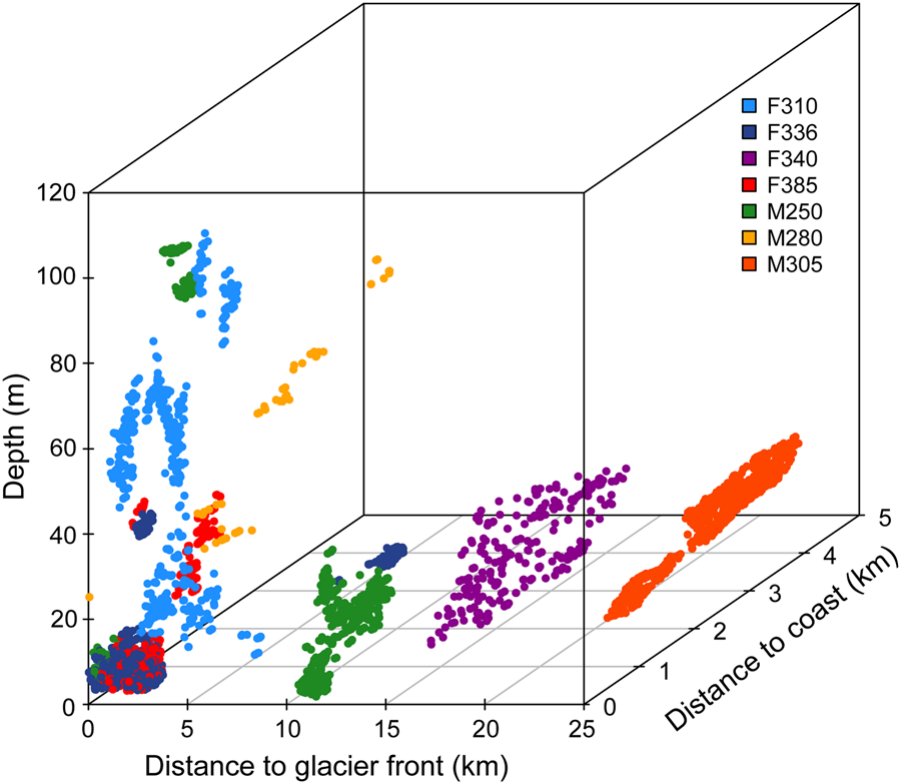
Distribution compared to key environmental variables. Top 25% of the kernel utilization distribution values of environmental space for seven adult bearded seals (*Erignathus barbatus*) equipped with GPS Argos CTD Satellite Relay Data Loggers in 2011–2012 in Svalbard, Norway.

There was considerable variation in habitat use among the individual bearded seals (Fig. 4). Generally, they all occupied shallow, coastal areas, including areas near tidal glacier fronts. However, some of the seals frequently used areas that were far from tidal glacier fronts or areas where the water was much deeper (Fig. 4). Some of the bearded seals changed their habitat use in relation to the environmental variables on a seasonal basis, but these changes were not always linear and the responses varied among individuals (Table 2). One seal (F385) increased its distance from both the coast and tidal glacier fronts and was in progressively deeper water from July to April (Table 2). Three other seals had seasonal changes in the depth of the water they occupied; F310 was in shallower water from July to April, F336 was in deeper water from July to April and M250 was in shallower water in the fall and winter and deeper water in the summer and spring. M250 also increased how far away it was from the coast from July to April (Table 2).

**Figure 5:**
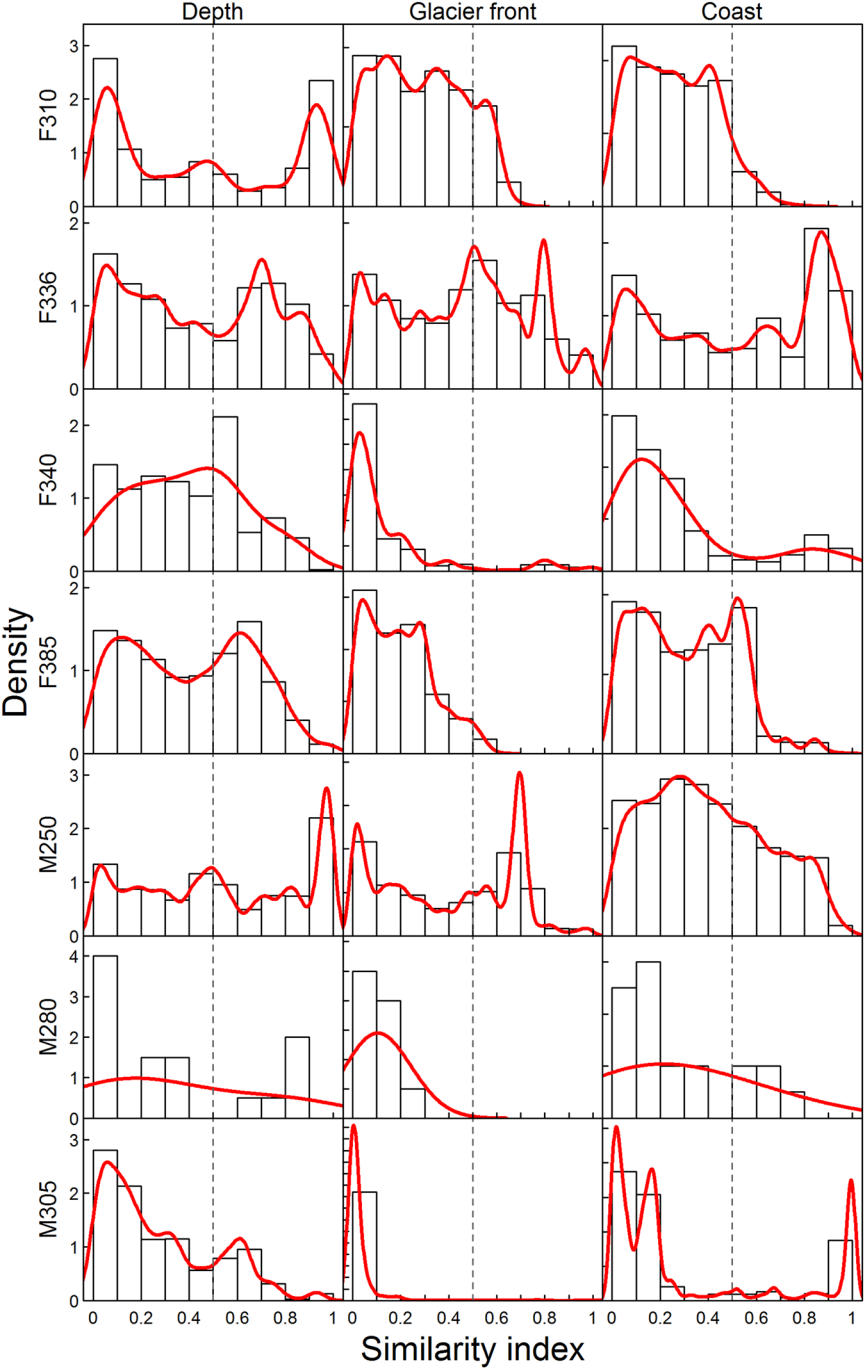
Environmental variable similarity plot. Similarity plot of the environment variables showing the level of specialization of the seals with respect to each environmental variable where 0 = specialist and 1 = generalist for seven adult bearded seals (*Erignathus barbatus*) equipped with GPS Argos CTD Satellite Relay Data Loggers in 2011–2012 in Svalbard, Norway. Values near zero indicate that an individual consistently used that environmental variable over the tracking period (the seal is found at similar distances from tidal glacier fronts or coastlines or is in areas that have a similar depth).

The level of specialization in the use of different habitats varied among the bearded seals (Fig. 5). Five of the seven seals were specialized in their use of glacier fronts (i.e. they inhabited areas that were similar distances to tidal glacier fronts throughout their tracking periods) and were tightly coastal in their distributions (Fig. 5). Two of the bearded seals selected areas with specific, and quite similar, bathymetric depths throughout their tracking periods (Fig. 5).

All of the bearded seals were found in areas with low sea-ice concentrations throughout most of their tracking periods. Between July and September, between 88–99% of locations were in areas with ≤ 10% sea-ice concentration. This fraction decreased to between 73–75% of locations in October and November, with ~ 25% of locations in these months located in areas with 10–40% sea ice cover. This trend continued in December and January when ~ 50% of locations were found in areas with ≤ 10% sea-ice concentration, while 43–46% of locations were in areas with somewhat more ice (10–40% sea-ice concentration). From February to April approximately half of the locations registered (44–56%) were in areas with 10–40% sea-ice concentration, but approximately 20% of locations registered during these months occurred in areas with higher sea-ice concentrations (ranging from 40–90%). A few locations (1–2%) registered during the winter (from December through to April) occurred in areas with land-fast ice.

### Activity patterns and diving behaviour

The bearded seals spent 74 ± 3% (mean ± SD) of their time diving, 21 ± 2% of their time resting or swimming at the surface and 5 ± 2% of their time hauled out. The proportion of time spent diving increased and the time spent at the surface and hauled out decreased from July to April, although the magnitude of these changes varied among seals (Table 2). Mean and maximum haul-out durations were 6.0 ± 1.9 h and 24.5 h, respectively. The mean interval between haul-out events was 120.8 ± 68.2 h. The interval between haul-out events increased with the onset of autumn for most seals, from 2.3 ± 1.5 d in August to 17.1 ± 13.6 d in March (mean ± SD, Table 2). One seal (M305) spent 145 d continuously in the water without hauling out (7 September 2012 to 30 January 2013). Thirty-two intervals between haul-out events were longer than 10 d and 14 intervals were longer than 20 d. Although these long intervals were spread throughout the tracking period, most took place during the winter (> 60% between December and March). However, there was no seasonal trend in the duration of haul-out events performed by individual seals.

Most of the diving done by the bearded seals was shallow and of short duration. The mean (± SD) and maximum dive depths were 24 ± 7 m and 391 m. Fifty percent and 95% of dives were shallower than 16 m and 70 m, respectively. The mean (± SD) and maximum dive durations were 6.6 ± 1.5 min and 24 min, and 50% and 95% of dive durations were shorter than 7.0 min and 12.4 min, respectively. Dive duration increased from July to April for all of the bearded seals. Dive depth and surface duration increased for most of the seals from July to April, though one of the five seals (dive depth: F310, surface duration: M250) did not show this general pattern. The remainder of the dive variables did not show any seasonal trends (Table 3).

**Table 3:** Dive parameters (mean ± 95% CI) for seven adult bearded seals (*Erignathus barbatus*) equipped with GPS Argos CTD Satellite Relay Data Loggers in 2011–2012 in Svalbard, Norway. The p-values (in bold) are the results of linear models testing for a seasonal trend in each variable.

Seal	Month	Dive duration (min)	Dive depth (m)	Surface duration (s)	Proportion of benthic dives (%)	Dive time at bottom (%)	Dive time in ascent (%)	Dive time in descent (%)	Same water mass at surface and max dive depth (%)
F310	All	7.3 (7.1–7.5)	34 (32–35)	91 (89–93)	51 (47–56)	43 (42–44)	29 (28–29)	17 (16–17)	67
	Jul	5.6 (4.4–6.8)	28 (21–35)	78 (68–87)	35 (18–53)	48 (41–54)	30 (25–34)	16 (13–20)	69
	Aug	6.4 (6.0–6.9)	43 (38–47)	97 (91–102)	48 (34–61)	55 (52–58)	24 (22–26)	14 (13–15)	52
	Sep	5.4 (4.9–5.8)	37 (33–41)	75 (68–83)	46 (27–65)	36 (34–39)	33 (31–35)	20 (18–21)	77
	Oct	6.1 (5.7–6.6)	28 (25–32)	97 (92–103)	49 (39–60)	37 (34–39)	29 (27–31)	19 (17–20)	69
	Nov	8.5 (7.9–9.0)	32 (29–36)	100 (93–106)	43 (32–54)	41 (38–44)	27 (25–29)	16 (14–17)	44
	Dec	8.4 (7.9–9.0)	22 (20–25)	92 (87–98)	40 (27–53)	40 (37–43)	31 (28–33)	16 (14–18)	77
	Jan	8.5 (7.9–9.0)	26 (23–29)	92 (87–97)	62 (50–74)	43 (40–46)	29 (27–32)	15 (13–17)	72
	Feb	7.0 (6.5–7.6)	32 (28–37)	83 (77–89)	75 (64–86)	44 (41–47)	29 (27–31)	17 (16–19)	64
	Mar	9.3 (8.7–9.8)	63 (54–72)	100 (96–105)	63 (43–83)	54 (51–57)	24 (22–26)	17 (15–18)	83
	*p*	**<0.001**	**0.359**	**0.019**	**0.002**	**0.203**	**0.650**	**0.404**	—
F336	All	6.1 (5.9–6.2)	15 (15–16)	85 (83–87)	87 (84–91)	51 (50–52)	23 (23–24)	15 (14–16)	84
	Jul	3.3 (2.5–4.0)	10 (7–12)	54 (46–62)	45 (12–78)	35 (30–41)	34 (30–38)	20 (16–23)	89
	Aug	6.6 (6.0–7.1)	10 (8–11)	74 (68–81)	91 (83–100)	55 (51–59)	18 (16–21)	13 (10–15)	99
	Sep	6.7 (6.3–7.1)	14 (13–16)	83 (78–89)	98 (95–100)	79 (77–82)	10 (9–11)	6 (5–7)	100
	Oct	5.7 (5.3–6.2)	10 (8–11)	84 (76–92)	92 (85–99)	40 (36–43)	26 (23–28)	17 (15–19)	99
	Nov	5.1 (4.6–5.5)	7 (6–8)	83 (73–94)	97 (94–100)	35 (31–38)	30 (27–33)	19 (17–22)	85
	Dec	5.2 (4.7–5.7)	8 (6–9)	90 (81–99)	95 (92–98)	38 (34–41)	29 (26–32)	18 (16–21)	95
	Jan	5.2 (4.8–5.7)	13 (11–15)	84 (76–92)	64 (45–82)	32 (30–35)	32 (29–34)	23 (20–25)	90
	Feb	6.2 (5.6–6.7)	22 (19–24)	83 (77–89)	66 (49–82)	39 (36–42)	31 (29–33)	19 (18–21)	72
	Mar	7.7 (7.3–8.2)	27 (24–29)	92 (87–98)	93 (88–97)	61 (58–64)	19 (17–21)	13 (11–14)	61
	Apr	7.3 (6.8–7.7)	31 (28–35)	102 (95–108)	94 (90–98)	70 (67–73)	16 (14–18)	9 (8–10)	46
	*p*	**<0.001**	**<0.001**	**<0.001**	**0.898**	**0.817**	**0.009**	**0.129**	—
F340	All	7.7 (7.4–7.9)	24 (22–26)	87 (82–93)	94 (90–98)	76 (74–78)	12 (11–13)	6 (6–7)	96
	Aug	7.5 (7.0–8.1)	32 (28–37)	82 (74–91)	93 (87–99)	70 (66–74)	16 (14–19)	9 (7–10)	88
	Sep	7.8 (7.4–8.1)	22 (21–24)	85 (78–92)	98 (96–100)	82 (79–84)	9 (8–10)	5 (4–6)	100
	Oct	7.6 (7.1–8.2)	17 (16–19)	98 (85–112)	90 (78–100)	75 (70–80)	11 (9–13)	5 (4–7)	100
F385	All	7.3 (7.2–7.5)	25 (24–26)	92 (90–93)	91 (88–93)	66 (65–67)	16 (15–17)	11 (10–11)	73
	Jul	5.5 (4.0–7.0)	11 (6–16)	75 (63–88)	32 (23–41)	37 (30–44)	37 (32–42)	23 (19–27)	97
	Aug	6.5 (6.1–6.9)	17 (15–19)	85 (81–89)	88 (80–97)	63 (60–65)	17 (15–18)	11 (10–12)	100
	Sep	6.8 (6.4–7.1)	18 (16–20)	91 (86–96)	97 (94–100)	66 (63–69)	14 (12–15)	10 (9–12)	99
	Oct	6.8 (6.4–7.2)	14 (12–16)	98 (90–106)	92 (87–98)	58 (54–62)	19 (17–22)	12 (10–14)	89
	Nov	7.7 (7.3–8.0)	22 (19–25)	84 (80–87)	88 (80–97)	63 (60–66)	16 (15–18)	11 (9–12)	56
	Dec	8.5 (8.1–8.9)	30 (27–33)	96 (93–99)	94 (87–100)	72 (70–75)	13 (11–14)	8 (7–10)	53
	Jan	8.7 (8.3–9.1)	32 (30–34)	96 (93–99)	93 (89–97)	72 (70–75)	14 (13–16)	8 (7–9)	93
	Feb	6.6 (6.1–7.1)	26 (23–29)	89 (82–95)	72 (57–87)	59 (56–63)	21 (19–23)	13 (12–15)	49
	Mar	7.6 (7.2–7.9)	35 (32–37)	92 (89–94)	92 (85–99)	73 (70–76)	13 (12–14)	11 (9–12)	56
	Apr	7.0 (6.6–7.3)	37 (34–39)	105 (102–109)	95 (91–100)	72 (70–74)	15 (14–17)	10 (9–11)	39
	*p*	**<0.001**	**<0.001**	**<0.001**	**0.982**	**<0.001**	**0.014**	**<0.001**	—
M250	All	5.3 (5.1–5.4)	24 (22–25)	64 (62–66)	66 (60–73)	44 (43–45)	27 (26–28)	18 (17–19)	80
	Jul	3.6 (3.0–4.1)	21 (15–27)	66 (54–78)	26 (0–53)	40 (36–44)	32 (29–34)	19 (17–21)	89
	Aug	2.8 (2.5–3.1)	10 (9–11)	57 (52–62)	9 (0–18)	31 (28–34)	34 (32–37)	20 (18–21)	100
	Sep	4 4 (4.0–4.7)	18 (15–20)	60 (57–62)	73 (55–92)	40 (38–43)	28 (26–31)	18 (16–20)	98
	Oct	5.2 (4.8–5.5)	21 (18–24)	65 (58–71)	86 (77–94)	42 (39–46)	27 (25–30)	19 (16–21)	88
	Nov	4.9 (4.5–5.4)	13 (11–16)	70 (63–77)	78 (66–90)	44 (41–48)	24 (22–27)	20 (18–23)	65
	Dec	6.2 (5.8–6.5)	13 (12–15)	63 (57–70)	85 (77–93)	41 (38–44)	30 (27–33)	20 (18–22)	91
	Jan	5.5 (5.1–6.0)	18 (15–20)	69 (64–75)	88 (78–99)	48 (45–51)	21 (19–23)	15 (13–17)	97
	Feb	6.0 (5.4–6.6)	24 (21–27)	60 (55–65)	62 (37–87)	56 (52–61)	23 (20–26)	12 (10–14)	50
	Mar	6.9 (6.2–7.5)	50 (42–57)	68 (64–72)	77 (66–88)	54 (51–57)	24 (22–26)	17 (16–18)	57
	Apr	6.5 (5.8–7.3)	51 (43–60)	66 (59–73)	68 (51–86)	46 (42–49)	29 (27–31)	20 (19–22)	66
	*p*	**<0.001**	**<0.001**	**0.393**	**<0.001**	**<0.001**	**<0.001**	**0.229**	—
M280	Aug	6.3 (5.6–7.0)	38 (33–43)	75 (68–81)	85 (73–97)	66 (62–70)	20 (17–23)	11 (9–12)	56
M305	All	8.6 (8.5–8.8)	20 (19–21)	92 (90–93)	94 (91–96)	77 (76–79)	10 (10–11)	5 (5–6)	80
	Aug	5.9 (5.2–6.5)	14 (12–16)	69 (59–79)	65 (43–87)	46 (39–53)	31 (25–36)	13 (10–16)	79
	Sep	6.1 (5.7–6.6)	16 (15–17)	96 (87–104)	87 (73–100)	77 (73–81)	12 (10–14)	5 (4–6)	51
	Oct	7.4 (7.2–7.7)	15 (14–15)	87 (85–90)	100 (100–100)	83 (80–86)	7 (6–8)	3 (2–4)	78
	Nov	8.7 (8.4–8.9)	19 (18–20)	91 (87–94)	100 (100–100)	85 (82–87)	6 (6–7)	3 (2–4)	99
	Dec	9.6 (9.3–9.9)	21 (20–23)	95 (88–102)	99 (98–100)	78 (75–82)	8 (7–9)	5 (4–6)	91
	Jan	9.6 (9.2–9.9)	24 (22–26)	83 (80–87)	95 (92–99)	72 (69–76)	12 (10–13)	6 (5–7)	89
	Feb	9.3 (8.5–10.0)	26 (22–31)	97 (88–105)	80 (67–94)	74 (70–78)	12 (11–14)	8 (6–9)	95
	Mar	10.3 (10.0–10.6)	19 (17–20)	102 (98–106)	96 (93–100)	80 (77–82)	9 (8–10)	6 (5–6)	59
	Apr	9.7 (8.9–10.5)	21 (18–25)	98 (92–105)	97 (92–100)	79 (73–85)	8 (6–9)	4 (3–5)	84
	*p*	**<0.001**	**<0.001**	**<0.001**	**0.220**	**0.089**	**0.119**	**0.219**	—
Mean	All	6.6 (5.6–7.7)	24 (20–29)	84 (76–91)	81 (70–92)	61 (51–71)	20 (15–24)	12 (8–15)	77 (68–85)

**Figure 6:**
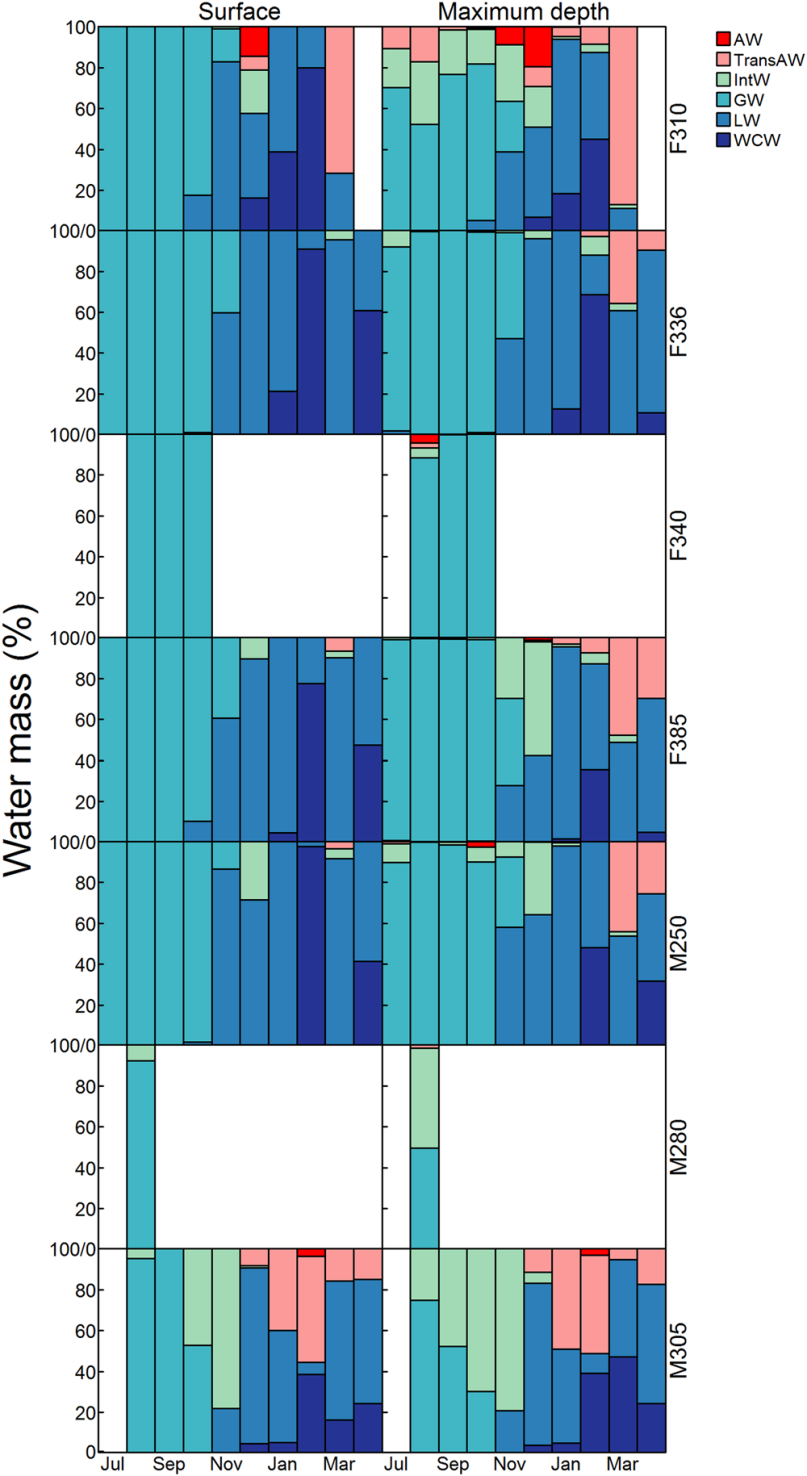
Water masses at the surface and maximum dive depths. Proportion of dives in different water masses at the surface (left) and at the maximum dive depth (right) by month for seven adult bearded seals equipped with GPS Argos CTD Satellite Relay Data Loggers in 2011–2012 in Svalbard, Norway. AW, TransAW, IntW, GW, LW and WCW stand for Atlantic Water (temp ≥ 3 °C; sal ≥ 34.65), Transformed Atlantic Water (1 ≤ temp > 3 °C; sal ≥ 34.65), Intermediate Water (temp ≥ 1 °C; 34 ≤ sal > 34.65), modified Glacial Water (temp ≥ 1 °C; sal < 34), Local Water (−0.5 ≤ temp > 1 °C; sal: any) and Winter-Cooled Water (temp < −0.5 °C; sal: any), respectively (modified from^97^).

The water masses encountered by bearded seals at the bottom of their dives, where they are assumed to do most of their foraging, varied seasonally and between individuals (Fig. 6). Although between 56 and 96% of the dives performed by individuals had the same water mass at the surface and maximum dive depth, a greater proportion of dives ended in a different water mass during the period February-April (Table 3). Water masses at maximum dive depths were dominated by a mixture of modified Glacial Water and Intermediate Water from July-October and a mixture of Local Water, Winter Cooled Water and Transformed Atlantic Water from December-April (Fig. 6). Atlantic Water and Transformed Atlantic Water were most common in the winter and spring months, and two of the seals dove into these water masses (F310, M305) more routinely than the other seals. These same two individuals also had more dives ending in Intermediate Water in the summer and autumn. During the winter/early spring, all seals dove into Transformed Atlantic Water while this water mass was largely absent at the surface. F310 (67%) and M280 (56%) had the largest difference between their surface and maximum depth water masses throughout the tracking period.

**Figure 7:**
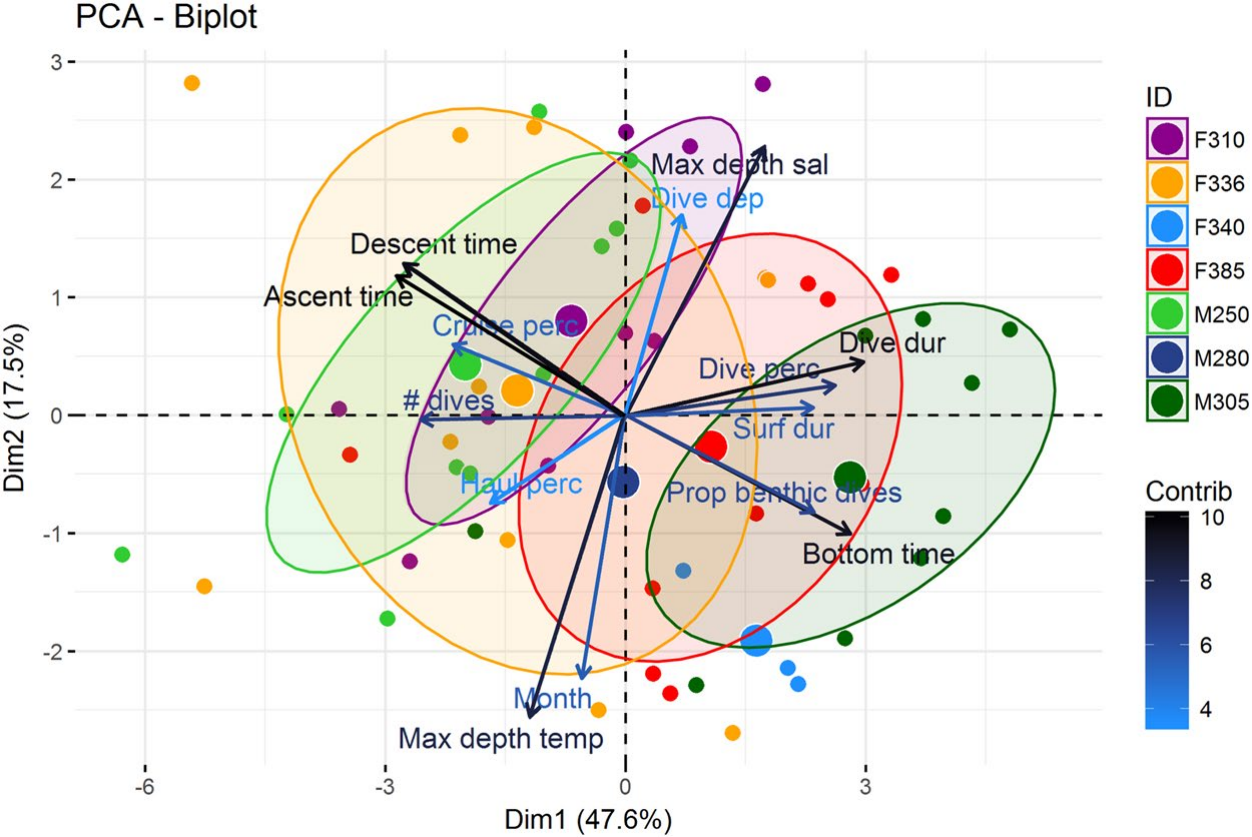
Dive variable PCA biplot. PCA biplot of the mean dive variables per month for seven adult bearded seals (*Erignathus barbatus*) equipped with GPS Argos CTD Satellite Relay Data Loggers in 2011–2012 in Svalbard, Norway. The colours of the dive variables indicate the contribution of that variable to the PCA. Seals F340 and M280 did not have enough monthly values for an ellipse to be drawn. Max depth sal, max depth temp, dive dep, dive dur, prop benthic dives, # dives, haul perc and cruise perc stand for salinity at the maximum dive depth, temperature at the maximum dive depth, dive depth, dive duration, proportion of benthic dives, number of dives in a 6 h period, percentage of time hauling out and percentage of time at the surface of the water, respectively.

A PCA biplot showed marked variation in diving behaviour among individuals, with 65.1% of the variation explained by the first two axes. The largest degree of individual variation occurred along an axis characterized by a greater percentage of dive time at the bottom, a longer dive duration, a longer surface duration, a greater percentage of time spent diving, a greater proportion of benthic dives, smaller proportions of dive time in ascent and descent and fewer dives (Fig. 7). The second main axis identified was a seasonal axis that included gradients in water temperature and salinity (Fig. 7).

## Discussion

This study is the first to report adult bearded seal movement and behaviour patterns covering most of the annual cycle. The bearded seals instrumented in this study exhibited high degrees of individual variability in their space use, dive behaviour, activity and movement patterns. There was a lack of seasonal patterns for many of the variables studied, with a few notable exceptions for some individual animals. Most seals displayed pronounced specializations in their habitat use and diving behaviour throughout their tracking periods.

The high level of individual variability in the behaviour of the bearded seals in combination with the small total sample size precluded the use of advanced statistical models to explore patterns. However, the high variability levels in behaviour documented in this study were similar to results reported for four adult females in the spring (May and June) pupping/maternal care period^20^, suggesting that the major findings of the current study are consistent with the limited available comparative data on this species. It is also noteworthy that the high level of variation in behaviour exhibited by the bearded seals is in contrast to other pinnipeds from Svalbard (i.e. ringed seals (*Pusa hispida*), walruses (*Odobenus rosmarus*), harbour seals (*Phoca vitulina*)), where adults exhibit more similar space use and diving patterns^30–34^. Young bearded seals in the Bering Sea also showed high levels of individuality in movement patterns, although habitat preferences were similar among individuals^17^. Some other pinniped species are also thought to display high levels of variation in their movement patterns (grey seals (*Halichoerus grypus*), Caspian seals (*Pusa caspica*))^35,36^. A larger sample size is needed to determine how many different “strategies” are employed by adult bearded seals, and if the level of individual specialization documented in Svalbard is unique to this region or is also present in other Arctic areas.

Although there was high inter-individual variation in habitat use and diving behaviour by the bearded seals in this study, the behaviour of individual animals was generally consistent (and specialized) over the tracking period. Whether the specialized habitat use and diving behaviour persists inter-annually deserves further study, although this will be challenging given how difficult it is to capture adult animals. The limited available data on bearded seals suggests that they show consistent inter-annual specialization in other behaviours, as mature females and mature males return to the same breeding areas from year to year and mature males repeat the same breeding strategy inter-annually^20,37,38^.

Bearded seals lose their hair diffusely throughout the year, but they do have a concentrated period of more intense moulting (replacement of the hair and upper layers of skin) in midsummer^10^. Moulting is less energetically costly if the animals are able to rest on the surface and circulate blood freely to the skin^39,40^. This likely explains the high percentage of time hauled out by some of the seals in July (> 20%) and the seasonal decrease in time spent hauled out from July to April. Overall, bearded seals spent little of their time (≤ 5%) hauled out. This is in contrast to some other pinnipeds in Arctic areas that haul out > 10% of the time (e.g. ringed seals, walruses, harbour seals, harp seal (*Pagophilus groenlandicus*))^41–44^, but is similar to a few other highly aquatic Arctic and Antarctic pinnipeds such as hooded seals (*Cystophora cristata*) and Ross seals (*Ommatophoca rossii*)^45–47^. In contrast to other ice-breeding species, nursing female bearded seals spend very little time hauled out (8 ± 3%) during the lactation period (ringed seals: 18%, harp seals: 29 ± 14%, grey seals: 72 ± 22%)^21,48–50^. The large dark-coloured female remaining in the water most of the time likely reduces the risk of polar bears finding the pup resting on the surface of the ice^21^.

Half of the bearded seals in this study (4 of 7) stayed within glacial fjords for the duration of their tracking periods, while the other animals were primarily found in shelf waters along the west coast of Svalbard. Two of these three seals foraged around the northern parts of Prins Karls Forland, an area that does not become ice covered even in winter. These animals travelled regularly into Kongsfjorden to haul out on calved pieces of glacier ice. Inner Kongsfjorden is the closest location that has active tidal glacier fronts that produce predictable ice haul-out platforms. This highlights the importance of hauling out, at least intermittently, given that these seals travelled at least 40 km each way to find suitable haul-out platforms. Edges of land-fast ice, drifting sea ice or calved pieces of glacier ice are used by bearded seals as haul-out platforms throughout the year^51^. Bearded seals do haul out on land^52^ (KMK & CL, pers. obs.), but this does not appear to be a common practice; hauling out on land was not documented in the current study.

One male bearded seal (M305) travelled a much greater distance than the other six instrumented animals. It travelled south from the capture location, along the west coast of Spitsbergen. The distances travelled by seals in Svalbard are much less than bearded seals in the Bering-Chukchi seas region, which undertake marked seasonal movements following the advance and retreat of seasonal sea ice^11,17^. However, the paucity of biotelemetry data over this species’ circumpolar range makes it difficult to conclude how sedentary vs migratory this species is in the various Arctic regions.

The 95% home range size varied markedly among the different bearded seals in accordance with their overall movement patterns. It was not directly related to length of the tracking periods of the individual animals, as a seal that only transmitted for 19 days had a larger 95% home range size than two seals that transmitted > 270 days. Given the large amount of variation in the 95% home range size, the 50% home range sizes were remarkably similar among the different seals. This suggests that bearded seals in Svalbard use a relatively restricted area to meet their core needs. In the spring, the two males had larger home range sizes than the three females that still transmitted locations. This is likely related to the breeding behaviour of some male bearded seals that use a “roaming” strategy^38,53^. Male bearded seals in Svalbard vocalize intensively from early April to mid July, with mating taking place after pups are weaned in mid to late May^54,55^.

The bearded seals in this study generally dove to shallow depths for short durations, with an average dive depth of < 40 m for all individuals. This seal species largely targets benthic prey, feeding on infauna, epifauna and benthic fish and invertebrates that live in shallow shelf areas^11–14,56^. The shallow diving done by the bearded seals in Svalbard is in accordance with the coastal bathymetry of this region. However, most of the seals in this study began diving to deeper depths and for longer durations during the winter and through into the spring. This is likely because the formation of land-fast ice pushes their distribution into somewhat deeper waters. Bearded seals generally do not travel into areas covered by land-fast ice, they remain along edges or occupy areas with drifting ice^13,16,17^. In other Arctic areas, bearded seals make and maintain breathing holes in thin land-fast ice, but this has never been documented in Svalbard^11,18^. The shift to deeper waters likely has consequences for their diet seasonally; females have been found to eat more pelagic fish and less benthic invertebrates in years when there is more extensive ice cover^57^. A potential diet shift, as well as an increased amount of dive time in ascent and descent, likely explains the increased dive duration in the winter and spring months compared to the summer.

In a diet study of female bearded seals from Kongsfjorden/Krossfjorden, large intraspecific variation was detected, with many of the seals found to be dietary specialists^57^. This finding is supported by the present study, where dive behaviour was quite specialized among the different seals over their tracking periods. Two of the seals in the current study did a lot of pelagic diving and spent less time at the bottom of their dives compared to the other seals. Bearded seals in Svalbard eat more fish (and fewer invertebrates) than bearded seals in other areas, with polar cod (*Boreogadus saida*) being the most frequent fish consumed^14^. Polar cod distribution varies with age class. Year classes one and two have sympagic distributions, inhabiting cracks and holes in sea ice, with older fish found both pelagically and benthically, with the largest fish found at the deepest depths^58–60^. Bearded seals targeting different year classes of polar cod, or differentially targeting fish vs benthic invertebrates could lead to differences in diving behaviour among individuals. Fjords on the west coast of Svalbard are currently undergoing rapid change, with Atlantic species becoming increasingly common^61,62^. Some seals (e.g. F310) appear to be specifically targeting these new prey types. Half of the diving done by F310 was pelagic diving, terminating in Atlantic Water or Transformed Atlantic Water consistently throughout the tracking period.

The almost continuous presence of some seals in areas near tidal glacier fronts indicates that bearded seals do not only occupy these areas to haul out. Bearded seals are likely not foraging to a great extent on benthos in these areas, due to the large amounts of sediment released from tidal glacier fronts^63,64^. A limited number of transmitted CTD profiles from bearded seals close to tidal glacier fronts showed sudden, negative salinity spikes, consistent with bearded seals ascending through glacial meltwater plumes. This suggests that bearded seals may be foraging on the predators (i.e. polar cod) of lower trophic prey trapped in these areas by in-fjord circulation patterns in the summer and early-autumn^51,65^. Foraging in front of tidal glacier fronts could explain the larger percentage of pelagic dives for some of the seals in July and August compared to later in the tracking period, as well as the presence of bearded seals in these areas year round. The occurrence of Arctic marine mammals near tidal glacier fronts throughout the year indicates that prey is plentiful in these areas^34,51^.

It is important to note that inaccuracies in bathymetric charts and in the estimates of locations of dive positions could result in some misclassification of benthic vs pelagic dives. Dive positions were calculated based on tracks made from an average of 15 GPS locations per seal per day. The high accuracy of GPS locations should minimize position error compared to Argos locations (36 m vs. 0.5–10 km)^66,67^, but bearded seals can travel large distances in two hours (maximum of ~6.5 km). Additionally, there is little bathymetry data for some areas in Svalbard, especially in areas near rapidly retreating tidal glacier fronts. However, the large variation in the percentage of pelagic vs benthic dives among individuals indicates that the differences observed are likely not solely due to misclassifications of dive type.

Possible age effects were not explored in this study because all of the tagged seals were adults (based on their body mass and worn state of their teeth, see^68,69^). Juveniles likely have different patterns of habitat use and diving behaviour compared to mature seals, especially during the spring breeding period. Further studies are needed on different age groups of bearded seals to assess how their movement, habitat, activity and diving behaviour changes over the course of development and to understand when bearded seals become specialized in these behaviours.

Climate change will likely be positive for bearded seal foraging and abundance in the coming decades in Svalbard. Ongoing declines in land-fast ice in Svalbard^28^ have already increased the distributional area for bearded seals. In 1994, bearded seal females were most often found in outer fjord areas in May and June, as continuous land-fast ice cover dominated inner fjord areas^20^. This is in marked contrast to current conditions in Svalbard. The bearded seals in this study (tagged in 2011 and 2012) used areas throughout the fjord, including the inner-most parts of fjords in the springtime. Adult female bearded seals in 1994 also dove to deeper depths in May than seals in the present study, likely because the more extensive land-fast ice cover in 1994 restricted them to deeper areas of the fjord^20^. Additionally, in the past more vocally active males were found when there was less ice cover, so decreased sea-ice extent could increase the proportion of mature males trying to breed and could also have consequences for the relative proportion of the “territorial” vs “roaming” breeding strategies^37^. The current impacts of climate change could also be positive for bearded seals’ foraging in Svalbard. Benthic biomass and productivity at shallow depths have increased due to less ice scouring and a longer growing season^70,71^. The increased distributional area and foraging conditions are possible explanations for why the bearded seals tagged in this study are heavier than those captured previously in Svalbard^68^. Tidal glacier fronts retreating onto land will also decrease the amount of sediment entering the water column, which will likely increase benthic biodiversity in the areas previously limited to low species diversity^63,64,72^.

The heavy exploitation of walruses in Svalbard that decreased their abundance from ~ 25,000 individuals in the 1600s to < 100 individuals in 1950 was likely positive for bearded seals in this region, as both species prey on benthic resources (and some walruses hunt bearded seals)^73^. The currently recovering walrus population might induce interspecific dietary competition between these two species^74^. Increasing competition from a recovering walrus stock was suggested to be a potential reason behind a temporal change in bearded seal diet in the Bering Strait a few decades ago^56,75,76^.

In the long term, climate change is likely to be more negative than positive for bearded seals (see^77^). Arctic ecosystems are predicted to shift from a sea-ice algae – benthos dominated system to a pelagic phytoplankton – zooplankton system^78^. Bearded seals in Svalbard currently consume large amounts of fish, so they may have enough dietary plasticity to meet their energetic needs in an altered food web. Studies from the Bering-Chukchi Sea region have shown that the diet of bearded seals is flexible and likely reflects prey availability^75,79,80^. However, reduced benthic prey biomass and increased competition from temperate pinnipeds (e.g. harbour seals) might become an issue for bearded seals in the future. Bearded seals do display considerable plasticity in other behaviours relevant to adjusting to climate change in the Arctic. During the last two decades, land-fast sea ice extents and the amount of drifting sea ice have declined markedly in Svalbard^28^. Small floes of sea ice broken off from land-fast annual ice was the preferred birthing, nursing and resting platform for bearded seals in Svalbard in the past^15^. But, bearded seals now use calved pieces of glacier ice for these activities in years when annual ice does not form in winter^51^. However, glaciers in Svalbard are in negative mass balance and the number and length of tidal glaciers are decreasing^72,81^. Lack of suitable ice habitat will likely be negative for bearded seal breeding success and long-term persistence of this species in the future^77^. Although bearded seals can haul out on land in areas with little sea ice, pupping and nursing on shore have not been documented for this species to date.

This study has shown that adult bearded seals in Svalbard display a high level of individual variability and population variability in their space use, diving behaviour, movement and activity patterns. Individual specialization is widespread across the animal kingdom and has important consequences for intra- and inter-specific interactions, population and community dynamics^8,9^. Having specialized strategies within a generalist population may also help a species adapt faster to changing conditions^3,9,82^. This may be especially relevant for Arctic marine mammals, as their long life-cycles decreases the chance of adaptation as environmental changes in this region are occurring extremely rapidly. The degree to which the variability documented in this study might help bearded seals survive in a rapidly changing Arctic environment, whether this individual variability is common for bearded seals in other Arctic regions and whether certain strategies are more successful than others are topics that warrant further investigation.

## Methods

### Instrumentation and data processing

Seven bearded seals were captured using various types of nets in the summers of 2011 (19 July-6 August, n = 5) and 2012 (14–18 August, n = 2) in Kongsfjorden (n = 6) and Krossfjorden (n = 1) in Svalbard, Norway (Table 1; Fig. 1). The seals were transferred into individual restraint nets where sex was determined by examination of the external genitalia and standard length (cm) and girth (cm) were measured. Body mass (kg) was calculated using the following equation (KMK and CL, unpublished data):

$$B={{\rm{e}}}^{-8.7167}\ast {L}^{1.0135}\ast {G}^{1.7686}$$

where *B* is body mass (kg), *L* is standard length (cm) and *G* is girth (cm). All seals were determined to be mature based on their calculated body masses and the worn state of their teeth^68,69^. A GPS Argos Conductivity Temperature Depth Satellite Relay Data Logger (CTD-SRDL; Sea Mammal Research Unit (SMRU), University of St Andrews, St Andrews, Scotland; http://www.smru.st-andrews.ac.uk/Instrumentation/Overview/) was glued to the hair centrally over the spinal column, anterior to the shoulder blades, positioned with the antenna facing posterior to maximize signal transmission and minimize drag with respect to the normal swimming posture of these animals. These tags transmitted: GPS locations; CTD profiles from the upcasts of selected dives (i.e. deepest dive in each 4 h period); dive data for individual dives (e.g. dive depth, dive duration); summary data for 6 h periods (e.g. percentage of the time diving, average dive duration); and haul-out data for individual haul-out events (e.g. start and end times for haul-out events) via the Argos satellite system (see^83^, http://www.smru.st-andrews.ac.uk/Instrumentation/Overview/, for more details). Animal handling protocols were approved by the Norwegian Animal Research Authority and the Governor of Svalbard and carried out in accordance with the relevant guidelines and regulations.

A continuous-time correlated random walk (crawl) model with a stopping model incorporated for the time spent hauled out was run on the GPS locations for each seal^84^. Hourly, dive, CTD and haul-out locations were extracted from the crawl models for each seal. All data exploration and analyses took place in R 3.3.3^85^.

### Home range

Dynamic Brownian bridge 50% and 95% home ranges were calculated for each seal by month and for the entire tracking duration, using the move package in R^86^. For the monthly models, a seal had to transmit data for at least 20 days in a given month to be included. This resulted in the exclusion of seven seal months. The inputs to the Brownian bridge movement models were: hourly locations; grid cell size of 500 × 500 m; location error of 50 m; window size of 35; and margin of 11 (see^86^ for more details).

### Habitat use

The distance to the nearest tidal glacier front (km), distance to the nearest coastline (km), bathymetric depth (m) and sea-ice concentration (%) were extracted for each location. Distances were calculated using the gDistance function in the rgeos package^87^. Shapefiles of the tidal glacier fronts (2001–2010) and coastline were retrieved from the Norwegian Polar Institute (www.npolar.no)^88^. A bathymetric data set of 100 × 100 m spatial resolution was created for western Svalbard using a combination of a 50 × 50 m spatial resolution bathymetric data set covering Kongsfjorden and Krossfjorden (Norwegian Polar Institute, www.npolar.no) and bathymetric data from the Norwegian Mapping Authority (https://kartkatalog.geonorge.no/). Sea-ice concentration data were obtained from the Norwegian Ice Service (Norwegian Meteorological Institute, http://polarview.met.no/).

The monthly mean and 95% confidence intervals for distance to the nearest tidal glacier front, distance to the nearest coastline and bathymetric depth were bootstrapped for each seal using 10,000 replicates (boot package^89^). Bootstrapping was used as it is a nonparametric method for calculating confidence intervals. Linear models were run for each seal and environmental variable to test for the presence of seasonal patterns. The response variables were included using the identity link and the Gaussian family was used to assess residual variation. The response variables were transformed, as necessary, to fulfil model assumptions. Day of the year (a running number with 1 indicating the first day of data transmission, July 19) was included as a predictor variable. Model evaluation took place by examining residual plots for homogeneity, homoscedasticity, normality and the presence of high Cook’s distance scores.

Utilization distributions in environmental space were made for each seal, to assess whether bearded seals used areas with similar environmental variables, by calculating a three-dimensional kernel density (kde function in ks package^90^; see^91^). Distance to the nearest coast (km), distance to the nearest tidal glacier front (km) and bathymetric depth (m) were the environmental variables used. The top 25% values of the 3D kernel were plotted for each individual to assess the level of similarity among seals in the habitat most frequently used.

Similarity indices for distance to the nearest coast (km), distance to the nearest tidal glacier front (km) and bathymetric depth (m) were made for each seal using foraging areas selected for each three-day interval^92^ to assess if seals were specialists or generalists in their habitat use. Hidden Markov models (HMMs) were run on crawl locations, selected every two hours for each seal using the moveHMM package^93^, to determine where foraging occurred. Proportion of the time diving and hauled out were included as covariates in the models in order to identify three states: travelling, foraging and hauling out. Both covariates were standardized before model inclusion. A density cluster was run on the forage locations identified by the moveHMM model for each seal in three-day intervals (dbscan function in dbscan package^94^). The eps value (i.e. size of the epsilon neighbourhood) for the dbscan function was chosen by finding the changepoint in the row means (cpt.mean function in changepoint package^95^) of the kNNdist function output. The closest foraging location to the centroid location of the density cluster with the most locations was selected as the foraging area for that three-day interval.

Each foraging area for a seal was compared to the other foraging areas for that seal and all foraging areas for all of the other seals. The differences in distance to the nearest coast, distance to the nearest tidal glacier front and bathymetric depth were calculated for each pair of points. The similarity index for each pair of points was calculated as the proportion of foraging areas from the other seals that had a smaller difference in the environmental value than the pair of points from a seal. Thus, low and high similarity index values indicate that the use of that environmental variable is specialized or generalized, respectively, for an individual (see^92^ for more details). Histograms of the similarity indices for each environmental variable were made for each seal.

### Activity patterns and diving behaviour

Dives by the bearded seals in this study were classified as benthic or pelagic dives (bottom depth of the dive vs bathymetry being the determinant). However, the bathymetry of the region is poorly resolved so many of the dive depths of the seals exceeded the mapped depths. Ninety percent of the dives that were deeper than the bathymetric depth were within 20 m of estimated depth, so in this study dives that were within 25 m of the bottom, or those that were deeper than the registered bathymetric depth at that dive location were classified as benthic dives with the remainder of the dives classified as pelagic.

The monthly mean and 95% confidence intervals for each activity and dive variable were bootstrapped in the same manner as for the environmental variables. The variables examined were: (1) dive duration (min); (2) surface duration between dives (s); (3) dive depth (m); (4) proportion of benthic dives (%); (5) proportion of dive time spent at the bottom (≥ 80% of maximum dive depth); (6) proportion of dive time spent in ascent and descent (%); (7) proportion of the time spent diving, at the surface of the water column and hauling out (%); (8) haul-out duration (min); and (9) time between haul-out events (h). To test for the presence of seasonal patterns, linear models were run for each dive and activity variable for each seal in the same manner as for the habitat variables (see above). More advanced statistical modelling was conducted (i.e. linear mixed-effect models (LMEs), and generalized additive mixed-effect models (GAMMs)) that included individual as a random effect. However, the results of these models are not presented herein as large individual variability for most of the variables in combination with a low sample size were deemed to produce results that did not reflect “reality.” GAMMs with random slopes for each individual (mgcv package^96^) were also investigated, but these were also deemed inadequate in representing the actual seasonal patterns for the individual seals.

Temperature and salinity were assigned to the surface (i.e. 1.5 m) and maximum dive depth of each dive using the CTD profiles collected by the seals using a three-way weighted average that incorporated time, distance and depth. This means that CTD profiles that were closer to a dive (i.e. in terms of time, distance and depth) were given greater weight than profiles further away when assigning water mass characteristics. The water mass at the maximum dive depth was investigated as seals generally are believed to forage at the bottom of their dives (for U shaped dives e.g. dives with significant proportions of bottom time). The western coast and fjords of Svalbard have dynamic oceanography both intra-annually and spatially due to variable, intruding Atlantic Water, glacial meltwater and seasonal cooling and warming^97^. The following six water masses were assigned to each surface and maximum dive depth using the temperature and salinity ranges defined in^97^: Atlantic Water (AW; temp ≥ 3 °C, sal ≥ 34.65); Transformed Atlantic Water (TransAW; 1 ≤ temp > 3 °C, sal ≥ 34.65); Intermediate Water (IntW; temp ≥ 1 °C, 34 ≤ sal > 34.65); modified Glacial Water (GW; temp ≥ 1 °C, sal < 34); Local Water (LW; −0.5 ≤ temp > 1 °C, sal: any); and Winter-Cooled Water (WCW; temp < −0.5 °C, sal: any). The water mass referred to as Surface Water in other papers^97^ is called modified Glacial Water herein.

To examine individual variability in dive behaviour, a principal component analysis (PCA) was run on the monthly mean of each dive variable for each seal. Variables included were the dive variables listed above, number of dives in a 6 h period, and temperature and salinity at the maximum dive depth.

## Data Availability

Data is available at the Norwegian Polar Data Centre. (doi: 10.21334/npolar.2018.3ef73637).
